# A Qualitative Analysis of Letters to Successors to Evaluate Medical Students’ Experience of a Mandatory Research Project

**DOI:** 10.1007/s40670-025-02516-3

**Published:** 2025-11-13

**Authors:** Declan Rosborough, Rajneesh Kaur, Joanne Hart

**Affiliations:** School of Medicine, Faculty of Medicine and Health, University of Sydney, Sydney, Australia

**Keywords:** Medical education, Research projects, Self-directed learning, Research education

## Abstract

**Background:**

Research capability is a key graduate outcome in medical education, yet student experiences of mandatory research components remain underexplored. This study investigated medical students’ experiences of a compulsory research project within an Australian medical degree program, identifying enablers, barriers, and perceived outcomes.

**Methods:**

A mixed-methods study was conducted using survey data from two student cohorts (2022 and 2023) following the completion of a 14-week research block. Quantitative data described participant characteristics, project types, and grades. Qualitative data, drawn from reflective “letters to successors”, were thematically analysed using iterative coding and external validation.

**Results:**

A total of 410 responses were analysed (74% response rate). Five core themes were identified: collaborative learning and support, skills and knowledge development, positive and negative project outcomes, autonomy, and professional skill development. Positive experiences were linked to project alignment with student interests and accessible supervision. Barriers included limited supervisor availability, social isolation, and time constraints. Many students valued the opportunity to develop research and transferable skills, with some reporting intentions to publish their work.

**Discussion:**

Student satisfaction was influenced by interest, support, and project relevance. Autonomy and skill development emerged as key enablers, while isolation and supervisor inaccessibility were common barriers. These findings highlight the importance of structured support, protected time, and student-centred project selection in enhancing engagement and ensuring high-quality, equitable research education in medical programmes.

## Introduction

The practice of evidence-based medicine requires basic research, literature searching, and critical analysis skills [8, 9]. In responding to this need and developing these skills, medical education programs are continuing to integrate and expand research education within their programs [6–8, 20, 26]. It is reported that barriers for students in developing these research-related skills include lack of available or protected time [7, 11, 13, 19, 25], insufficient available research projects [11, 16, 25], and inadequate supervision, training, and support [7, 14, 16, 19, 28]. These factors contribute to variability in the outcomes of research skill development [19].

These barriers are underpinned by gaps in clearly defined learning outcomes and the absence of benchmarked programs within a consistent framework [9]. While both the Australian Qualifications Framework Level 9E [2] and the Australian Medical Council [1] require graduates to demonstrate an understanding of research, the expected outcomes are not explicitly articulated. Instead, they are framed in broad terms, such as applying scientific methodology to research questions, identifying appropriate study designs, and complying with safety frameworks, legislation, and clinical guidelines. The lack of specificity leaves considerable room for interpretation, resulting in variability across institutions and programs. This variability can lead to inconsistent skill acquisition, with some students gaining strong research capabilities while others graduate with only a minimal understanding. Greater clarity and benchmarking of outcomes could support more consistent integration of research training and ensure that all graduates achieve a baseline level of research competence.

Inconsistent expectations around research in medical education can foster detrimental perceptions, such as viewing research as optional [9], questioning its relevance to clinical practice [20], or missing opportunities to develop skills fundamental to medical practice [6]. Evidence from mandatory research programs shows that when these gaps are addressed, students gain stronger research literacy and critical analysis skills [19, 27]. Understanding the diversity of student experiences in such programs, including both enablers and barriers to research skill development, can inform the design of future curricula and support improved graduate outcomes.

While previous studies have examined student outcomes from research programs, including publication rates [5, 32], career trajectories [10, 24], and skill acquisition [4, 35], less attention has been paid to the lived experiences of students, particularly in mandatory research settings. Reflective narratives offer valuable insight into how students navigate research projects, what enables or hinders their engagement, and how these experiences shape their perceptions of research and academic practice [22, 23]. Moreover, existing literature rarely examines how students’ personal reflections on supervision, support, autonomy, and relevance inform their overall satisfaction and learning.

This study focuses on understanding the experiences of medical students within a mandatory research program through analysing student responses, in the form of “letter to successor” style reflections [17]. Some studies have addressed student experience within research programs [7, 28]; however, there is less research utilising examination of student reflections. As such, this study aims to gain a broader understanding of the student experience of the research program and answer:

1. What are the student experiences and outcomes of a mandatory research project?
2. What are the challenges and facilitating experiences within a mandatory research project?

The outcome of this analysis will be used to inform the future design and implementation of research projects within the medical curriculum and ensure that students are adequately prepared for future research and evidence-based practice.

## Methods

### Study Context

The University of Sydney 4-year graduate-entry Doctor of Medicine Degree (MD) enrols students from a wide range of academic backgrounds (not limited to those with a biomedical science undergraduate degree) and with various prior research and employment experiences. The MD Project is a mandatory, individual research project of 14-week full-time duration. Students receive 40 h of training in research methods, statistics, and research ethics education in preparation [19]. Through experiential, project-based learning, students develop research skills and other professional skills such as self-motivation, time management, and collaboration within research environments. They undertake hands-on projects across a wide range of fields, including clinical studies, biomedical science, public health, medical education, bioinformatics, and health policy. Project progress is monitored through milestone assessment tasks, culminating in a 3000-word scientific report. The learning outcomes and milestone tasks for the MD Project are shown in Tables 1 and 2. While students are encouraged to publish their work or present it at conferences, this is not mandatory. Research project supervision is provided by academic staff or university-affiliated researchers, though supervisors are not required to have formal supervision training or receive remuneration, and no project funding is available. This is one of the largest research project programs of its type in Australia, with an annual cohort of 250–300 students, 250+ supervisors, and projects located across 10 locations in metropolitan Sydney and rural New South Wales, Australia.

**Table 1:** Learning outcomes

Research skills	Professional skills
Formulate research questions	Communication and interpersonal skills
Ethical judgment and professional integrity in research	Time management and organisation
Literature search and review	Teamwork and collaboration
Research design and methodology	Critical thinking
Data collection and analysis	Resilience and adaptability
Scientific reporting	

The learning outcomes are split into research skills and professional skills, which are both developed during the research project.

**Table 2:** Assessment tasks

Milestone	Task
1	Research project proposal and plan Appraisal of ethical issues pertaining to the project
2	Literature search strategy and literature review
3	Final project plan, including data analysis plan and progress report
4	Draft results section
5	Draft of final report
6	Final oral presentation
7	Final written report

Assessment tasks are set out as milestones that are designed to progress the students through their research projects.

### Theoretical Framework

The student evaluation task was informed by Schön’s theory of reflective practice, which emphasises the importance of reflection-in-action and reflection-on-action as mechanisms for professional learning and development [31]. To elicit students’ reflective insights on their experiences during the research block, we employed a structured reflective task in the form of “letters to successors.” [17]. This approach invited students to write a letter to future cohorts, offering advice and reflections based on their own experiences.

The “letters to successors” format was chosen as a reflective tool because it allows students to provide authentic, personal insights based on their direct experiences. This type of reflection encourages deeper engagement, as students are writing with the intent to help future students, which often leads to more honest, candid feedback on both positive and negative aspects of their learning. By framing their reflections as advice to others, students are more likely to think critically about their learning processes, challenges, and achievements, offering valuable guidance that can inform program improvements. Further, this approach promotes metacognitive thinking, where students not only reflect on what they did but also on how they did it and how it can be done better by others. This method has been shown to foster a sense of responsibility and ownership over the learning experience, contributing to the development of important professional skills such as critical thinking, self-assessment, and peer guidance [30].

### Data Collection

Data were collected in an online pre-post survey. At the commencement of the MD Project, students were asked to provide prior research experience data and complete a self-assessment of their research skills [33]. The online post-survey was administered at the submission of their scientific report, but before grades were published. Survey responses were identifiable, but deidentified prior to analysis. The post-survey also contained the same self-assessment of their research skills and collected information about their project and research topic and two “letter to successor”-style questions:

1. What were the three most important things you learned from doing your MD Project?
2. What advice would you give to future students embarking upon the MD Project? Include in your answer a reflection on the three best and three worst aspects of the MD Project (up to 200 words).

Student responses were declared as mandatory; however, they were not assessed nor monitored for completion. Further data on student grades and enrolment data (gender, local or international student status, MD Project grades) were obtained from student records.

### Data Analysis

Participant characteristics data were analysed using descriptive statistics to summarise participant characteristics, including measures such as frequencies, percentages, median, and IQR, as appropriate. This provides an overview of the sample composition in terms of variables such as gender, student status (local or international), grades, and prior research experience.

We conducted a thematic analysis informed by a content analysis approach to identify and categorise recurrent patterns within the data. Our process followed the structured steps outlined by Braun and Clarke [3] with an emphasis on transparency and consistency in coding. First, we familiarised ourselves with the data through repeated reading of transcripts. Second, we generated initial codes based on salient features relevant to the research questions, using primarily semantic and descriptive coding focused on explicit content. Third, we collated these codes into potential themes, and fourth, we reviewed the themes by checking them against both coded extracts and the dataset as a whole. Fifth, we defined and named themes according to their conceptual coherence and relevance to study objectives. Finally, we produced the written analysis, ensuring alignment between data extracts, analytic claims, and research questions. This process was collaborative and systematic, with regular team discussions to refine coding decisions, resolve discrepancies, and confirm reliability.

In addition to the primary thematic analysis, we conducted a comparative sub-analysis to explore whether specific participant characteristics were associated with variations in qualitative responses. This included examining potential differences in perceptions of satisfaction and learning according to prior research experience, as well as exploring whether project grade was related to the nature or tone of student reflections. Transcripts were reviewed with these variables in mind, and illustrative differences in content or emphasis were noted and compared across relevant subgroups. This analysis remained exploratory and was intended to identify potential patterns rather than test predefined hypotheses. Data management and coding were facilitated using nVivo v14 (Lumivero, Denver, CO) allowing for consistent application of the coding framework across cases.

## Results

### Participant Characteristics

Participant characteristics are presented to contextualise the sample and provide an overview of its composition. These data offer insight into relevant attributes such as cohort size, gender, enrolment status, and prior research experience, and are summarised using descriptive statistics. A total of 557 student survey responses were collected from the 2022 and 2023 cohorts following completion of their research project, representing an overall response rate of 75% across both year groups. Response rates by cohort are presented in Table 3.

**Table 3:** Response rates to survey

Year	Responses (*n*)	Cohort size (*n*)	Response rate (%)
2022	224	253	89
2023	186	304	61
Total	410	557	74

Participant characteristics for the 2022 and 2023 cohorts are summarised in Table 4. The gender distribution was relatively balanced across both cohorts, with a slightly higher proportion of male students in 2022 and female students in 2023. Most students were enrolled as local students, with a notably lower proportion of international students in 2023 compared to 2022, likely a result of COVID-related border access to Australia for international students. Across both cohorts, a substantial proportion reported prior research experience, most commonly through an undergraduate research project or an undergraduate Honours degree. Relatively few students reported having completed a Master’s, PhD, or Post-doctoral qualification. Most participants indicated no prior research-related employment.

**Table 4:** Participant characteristics

Cohort	2022 *n* (%)	2023 *n* (%)
Total students (***n***)	253	304
Gender		
Female	112 (44.3)	142 (46.7)
Male	140 (55.3)	159 (52.3)
Not indicated	1 (0.4)	3 (1.0)
Student enrolment status		
International	49 (19.4)	17 (5.6)
Local	204 (80.6)	287 (94.4)
Prior research experience		
Undergrad research project	96 (37.9)	114 (37.5)
Bachelor with Honours	50 (19.8)	51 (16.9)
Masters	17 (6.7)	17 (5.6)
PhD	4 (1.6)	5 (1.6)
Post-doctoral studies	2 (0.8)	1 (0.3)
No prior research experience	70 (27.6)	111 (36.5)
No Data	10 (4.0)	0 (0.0)
Prior research employment		
Yes	33 (13.0)	30 (9.9)
No	210 (83.0)	274 (90.1)
No data	10 (4.0)	0 (0.0)

Details of MD Project types, research focus, and grades are summarised in Table 5. Across both cohorts, most students completed a scientific report, with smaller proportions undertaking various forms of literature reviews, study protocols, or product development projects. The most common research type was clinical research, followed by laboratory-based projects and public health or epidemiology research. Projects in medical education and big data or informatics were less frequently taken. Project grades ranged from 37 to 93 out of 100, with a consistent median grade across cohorts, suggesting comparable performance over time.

**Table 5:** MD Project details

Cohort	2022 *n* (%)	2023 *n* (%)	Total *n* (%)
MD Project type			
Scientific report	175 (69.2)	201 (66.1)	376 (67.5)
Narrative review	16 (6.3)	22 (7.2)	38 (6.8)
Systematic review	31 (12.3)	35 (11.6)	66 (11.9)
Scoping review	12 (4.7)	28 (9.2)	40 (7.2)
Study protocol or ethics approval project	5 (2.0)	10 (3.3)	15 (2.7)
Product development project	14 (5.5)	8 (2.6)	22 (3.9)
Research type			
Clinical research	154 (60.9)	167 (54.9)	321 (57.6)
Laboratory project	32 (12.6)	47 (15.5)	79 (14.2)
Big data or informatics research	5 (2.0)	12 (3.9)	17 (3.1)
Public health or epidemiology research	47 (18.6)	64 (21.1)	111 (19.9)
Medical education	15 (5.9)	14 (4.6)	29 (5.2)
MD Project grade			
Range (of 100)	37–91	38–93	37–93
Median (IQR)	75 (68–81)	77 (70–82)	76 (69–81)

### Letters to Successors

Thematic analysis of the letters to successors data identified a series of patterns that reflect participants’ perspectives and experiences in relation to engaging in student research projects. The following sections present the key themes derived from the data, supported by illustrative quotations. Each theme is described in detail, highlighting both commonalities and points of variation across participants’ accounts.

1. Collaborative learning and support mechanisms

The most frequently discussed aspects of this theme were around support, with as many students discussing feeling supported as those who discussed a lack of support. Most commonly, students felt supported by supervisors and research teams who were able to guide them during the work.

Conversely, a lack of support came from poor access to supervisors, often due to unavailability and difficulty in accessing university resources, such as statisticians, in a time frame that fit within the research block. Consequently, many students advised future students to seek assistance early, seek a variety of support types, and meet with supervisors early and regularly.

"*I was EXTREMELY lucky to have the most kind, helpful, supportive and hardworking supervisor that made the project a breeze and so interesting*".—2022 student

"*The worst aspect was difficulty getting time with my supervisor. As a busy consultant, they were virtually unavailable for large sections of the project*"—2023 student

"*Be open to collaboration and seek advice and mentorship from all those who are available to you*"—2022 student

Given the nature of this project as a formative experience in developing basic research skills, these results were not surprising, with students finding their projects simpler to navigate while feeling supported. The ideas of developing communication and teamwork skills were identified, typically in the form of advice for future students to work cooperatively, noting that this discussion was overshadowed by discussions of support.

"*..the importance of active communication with my supervisor to ensure I receive appropriate feedback and guidance. A good relationship with my supervisor was beneficial during logistically difficult parts of the project*".—2022 student

2. Development of skills and knowledge

The two most frequently identified areas of skills and knowledge development were literature search and critical analysis skills as well as general research skills. Students identified that developing these skills and knowledge was an enjoyable experience and enabled greater motivation and confidence.

"*Getting to learn how research is done, this gave me new insight into analysing other research*."—2022 student

Some responses were more specific about skills gains, citing practical skills, skills in a range of software programs, statistics skills, writing skills, and medical speciality knowledge. These were highly dependent upon project type.

"*I learnt the process of conducting a systematic search strategy and using software (Rayyan) to help with screening*".—2022 student

3. Positive and negative project outcomes

Although sentiment was not explicitly coded, responses generally reflected a positive overall view of the project, though the underlying reasons for this varied.

"*I really appreciated the opportunity to experience the research process through, from conception of the idea to execution in the final write up. There was a real sense of accomplishment.*"—2022 student

"*Not engaging for students who do not have any interest in research.*"—2023 student

There was a significant appreciation for the MD Project as a whole, with students appreciating how the MD Project was run, the independence of the project, and having interest within the project topics. Typically, where negative positions were identified, they were in response to specific aspects of the project, i.e. personal motivation and time management or a lack of support, rather than the overall experience of the MD Project. Direct project benefits appeared as personal development, developing professional relationships, and identification of real-world application, through either adding to bodies of research or developing skills that could translate to evidence-based clinical knowledge.

"*Working within a small team to create something that is useful in the real world was great*."—2023 student

Tellingly, the data strongly indicate that feelings of social isolation and loneliness were common experiences among students undertaking the MD Project, particularly in the context of remote or independent work. Despite having supervision teams, many students described the project as “isolating at times”, especially when working from home during COVID-related restrictions. The extended period of independent work, “14 weeks doing independent work could get quite isolating”, was repeatedly noted as a challenge, with students often feeling disconnected from peers and the broader academic environment. Several students highlighted that “working separately from your peers” and “not seeing many people for most of the 14 weeks” contributed to reduced morale and motivation. Others emphasised the importance of proactive strategies to combat isolation, such as “staying in contact with other students”, “having group study sessions”, and “catching up with friends often”. The sense of loneliness was not limited to social disconnection but was often compounded by a lack of structured schedules and minimal feedback, making it difficult for students to stay engaged and self-motivated. Overall, while the MD Project fostered independence, the findings suggest a clear need for more structured opportunities for peer interaction and regular engagement with supervisors to mitigate the isolating nature of the research experience.

4. Autonomy

Barriers and negative experiences included limited project choices, dissatisfaction with how projects were structured or implemented, and challenges associated with working independently. These discussions spoke to difficulties in maintaining motivation and positivity throughout the project due to a lack of experience or obvious clinical connection, a dislike of the project process, social isolation, and perceived lack of interest in project topics. Conversely, as an enabling and positive experience, students enjoyed the flexible nature of the workload within the project and the ownership of the project.

"*The MD project was a great opportunity to have creative license over developing and executing clinical research. I really appreciated the opportunity to experience the research process through, from conception of the idea to execution in the final write up*".—2022 student

"*The worst aspect for me was initially being motivated to engage in the project - because of the lack of project options […] and the compulsory requirement to preference two projects from your home clinical school, I was allocated to one of the compulsory […] preferences*."—2022 student

Almost as direct supportive evidence of this, students provided advice in the form of adequate preparation for the projects, a focus on selection of project based on interest area, having specific motivations and goals, and having a positive and resilient attitude for the duration of the project. These ideas can all be directly linked to the barriers and negative experiences within this theme, providing a clear set of common problems and a set of solutions.

"*Pick a project topic that you are interested in and don’t think too much about its difficulty because if you are interested in the topic you will find yourself working on it effortlessly*."—2022 student

5. Professional skills development

Discussion of organisation and time management was common in student responses, emerging both as evidence of learning and development and as reflective advice for future students. Many students identified limited time as a barrier, not due to a lack of protected research time, but rather because the time available was insufficient to achieve meaningful results, which negatively impacted project outcomes and motivation. In contrast, a smaller number of students felt that too much time was allocated, typically expressing frustration over reduced clinical exposure and questioning the relevance of research within the medical programme.

"*One of the best and worst things about this block is the limited time. It means we can concentrate fully on the project and get the most out of the experience, but it also limits the scope of the research we can do. In saying that, there are always chances to continue working on the project in your spare time once the block is over.*"—2022 student

"*Regarding worst aspects; length of the project - I think we may have been given too much time.*"—2023 student

### Relationships Between the Participant Characteristics and Themes

A comparative sub-analysis was undertaken to explore whether specific participant characteristics were associated with differences in qualitative responses. This included examining potential variations in student satisfaction, perceived learning, and reflections on the research experience based on prior research experience (e.g. undergraduate research, Honours, or higher degrees), project grade, and enrolment status (local or international). Transcripts were reviewed for thematic differences across these subgroups; however, no clear or consistent patterns emerged. The nature, tone, and content of student reflections appeared broadly similar regardless of these background variables.

## Discussion

This study explored medical students’ experiences in a mandatory research project by analysing reflective “letters to successors” from two cohorts. Thematic analysis revealed five central themes: collaborative learning and support mechanisms, development of skills and knowledge, project outcomes, autonomy, and professional skills development. Students generally reported positive experiences, particularly when they had access to supportive supervision, worked on projects of personal interest, and developed relevant research skills. However, recurrent challenges, such as limited access to supervisors, constrained timelines, social isolation, and restricted project choice, negatively affected engagement and motivation for some students. Demographic and project-related variables, including prior research experience and project type, were not associated with differences in thematic responses, suggesting that these experiences were widely shared across the cohort.

### Student Experience and Satisfaction

Overall, students expressed a sense of satisfaction with the research project. Key drivers included intellectual engagement with the project topic, the opportunity to gain autonomy, and the development of skills seen as valuable for future clinical or academic roles. These findings build on prior research indicating that perceived relevance and ownership contribute significantly to student satisfaction [25], by showing that these drivers are not sufficient on their own; structural barriers such as inaccessible supervision and constrained timelines can erode motivation even when projects are relevant to students’ interests. However, satisfaction was tempered by a number of recurring barriers. Students who struggled to connect with their projects, faced supervisor inaccessibility, or encountered time constraints often reported reduced motivation and enjoyment. These findings reinforce that student satisfaction is shaped not only by the project’s academic content but also by structural and relational factors such as mentorship and perceived autonomy, suggesting that enhancing supervisory support and fostering student ownership may further improve both satisfaction and the educational value of research programs.

### Project Outcomes and Perceived Value

Students’ reflections on project outcomes were more strongly shaped by their experience of the research process than by specific achievements such as grades or publication. Many expressed a sense of accomplishment from completing a project and contributing to an area of interest, even where the research was not publishable or externally validated. Tangible outcomes, such as publications and conference presentations, were identified as valuable, with nearly half of the 2023 cohort reporting intentions to disseminate their work. These outcomes were associated with feelings of success and enhanced confidence in conducting research. However, others noted that limited time, complex projects, or minimal support impeded their ability to achieve meaningful outcomes. The factors identified here resonate with previous literature [7, 11, 13, 16, 19, 25], but our findings add nuance by illustrating how limited time and minimal institutional support act as mechanisms that constrain students’ ability to translate their research into tangible outputs such as publications or presentations. Overall, this highlights the importance of framing the research project primarily as a learning experience rather than a vehicle for academic output, while still supporting opportunities for dissemination where feasible.

### Role of Support and Collaboration

Supportive supervision and institutional resources were consistently identified as critical enablers of student success. Students described supervisors and research teams as valuable sources of guidance, with positive supervisory relationships enhancing motivation and project clarity. Conversely, a lack of access to supervisors, often due to clinical workloads, was one of the most frequently cited barriers. Students also reported difficulty accessing university services such as statistical support within project timelines. These findings align with prior literature identifying supervision quality as a key determinant of student research outcomes [7, 14, 16, 19, 28]. In response, many students advised their successors to engage early and consistently with support networks, highlighting the value of supervisor training and clear communication about available institutional support, which may help strengthen student research experiences.

### Impact of Autonomy

Autonomy emerged as both a benefit and a challenge. Students valued the flexibility and ownership the project afforded, which contributed to a sense of professional identity and capability. However, some struggled to maintain motivation in the absence of structured schedules. This has been reported before, and development of self-directed learning is an important element in research project-based learning [34]. Students had more problems with motivation when working on projects that were assigned rather than by choice, and this is in line with the literature [7]. The lack of alignment between student interests and allocated topics was a recurring theme, with several students reporting dissatisfaction with the nature of project selection processes. While independence is a desirable educational outcome, these findings suggest that autonomy is most effective when scaffolded through preparation, project relevance, and supportive supervision, and that providing greater student input into project selection may help sustain motivation and engagement.

### Social Isolation

A notable and unexpected finding was the prevalence of social isolation and loneliness during the research project. Students across both cohorts described working independently as isolating, with this particularly acute in the COVID-affected 2022 cohort, where substantial disruption of research activities and remote working exacerbated disconnection from peers [15]. However, the issue was not confined to the pandemic context. In our setting, the individualised structure of projects, limited protected research time, and variable supervisory access also contributed to feelings of isolation. These structural features, combined with minimal opportunities for structured interaction and feedback, reduced morale and self-motivation. Thus, social isolation appears to reflect both contextual effects of the pandemic and inherent characteristics of the project design. Students commonly recommended maintaining social contact, attending study sessions, and seeking collaborative opportunities as strategies to mitigate this challenge. This aligns with the literature on researcher well-being, which identifies isolation as a barrier to productivity and satisfaction [12, 29]. Our study extends this work by showing that isolation can stem not only from contextual disruptions such as the pandemic but also from the structural design of individualised projects, where limited peer interaction and supervisory access leave students vulnerable to disengagement. Taken together, these findings suggest that unless deliberate mechanisms for peer connection are embedded, research projects risk undermining student well-being and engagement, reinforcing the potential value of structured peer activities to foster community and accountability during the research block.

### Professional and Research Skill Development

Students reported substantial development of both research-specific and transferable skills. Core competencies identified included literature searching and appraisal, research design, data analysis, writing, and the use of specialised software. In addition, many students noted growth in time management, organisation, communication, and problem-solving abilities. These skills are foundational for both evidence-based clinical practice and future academic work [6] and their consistent identification suggests that the program effectively supported key learning outcomes. Nonetheless, students reported variability in skill development depending on project type, suggesting the need for greater clarity around core research competencies expected across all project formats. The development of a unified research competency framework could provide a more consistent educational experience and ensure that all students graduate with a baseline of research capability [6–9, 11, 21, 26]. Clarifying and standardising expectations through such a framework may help medical schools strengthen research training and support equitable outcomes across diverse projects.

### Influence of Participant Characteristics

The comparative sub-analysis revealed no significant associations between student characteristics (e.g. gender, enrolment status, prior research experience) and the emergent themes. Similarly, there were no differences in thematic content by project type, research type, or project grade. This suggests that key challenges and enablers, such as the importance of support, project alignment, and time constraints, were widely experienced. While one might expect prior research experience to enhance confidence or reduce perceived barriers, this was not evident. These findings speak to the value of employing universal design principles [18] in research education, ensuring that all students, regardless of background, are adequately supported.

### Applicability to Broader Educational Contexts

Although situated within an Australian medical program, these findings highlight themes with broader relevance to research education. Enablers such as supportive supervision, project alignment, and skill development, along with barriers including isolation, time constraints, and limited choice, have been reported internationally across medicine and allied health. This suggests that the experiences described here may be transferable to diverse educational systems. Nonetheless, the mandatory nature of the project and its integration into a compressed curriculum are context-specific features that may have amplified some challenges. Taken together, the results provide insights for programs seeking to embed mandatory or elective research training in ways that are both effective and equitable.

### Strengths

This study benefits from a large and well-defined sample, with high response rates across two consecutive cohorts. The qualitative data provided rich, authentic insight into student experiences and enabled the identification of meaningful patterns across diverse projects. The thematic analysis was strengthened by iterative coding and external review, supporting analytical rigour. Moreover, the insider perspective of the student researcher provided valuable contextual insight, particularly when interpreting program-specific language or brief responses, thereby enhancing the relevance and authenticity of the findings.

### Limitations

Several limitations should be considered when interpreting these findings. The dual role of the student researcher introduces potential subjectivity, which may have influenced theme interpretation despite mitigation through external review and iterative coding [3]. The brevity of responses, constrained by word limits, likely limited the depth of qualitative insights, while the mandatory nature of the survey may have encouraged more cautious reflections. In addition, the sample size precluded subgroup analyses, restricting exploration of intersectional experiences. These factors suggest that some nuances of student perspectives may not be fully captured.

### Future Directions

Future studies could address these limitations by using independent coding teams, richer qualitative methods such as interviews, and voluntary, anonymous participation to encourage candour. Larger, multi-institutional samples would also allow subgroup analyses and strengthen generalisability. Longitudinal research is needed to examine the lasting impact of research experiences on clinical practice and academic engagement. At a programe level, priorities include enhancing supervisor preparation, increasing student choice in project selection, mitigating isolation through structured peer support, and developing a core competency framework to ensure consistent learning outcomes.

## Conclusions

This study provides a comprehensive analysis of student experiences in a mandatory research project within a medical program. While students generally reported satisfaction and recognised the academic and professional value of the project, several areas for improvement were identified. Key enablers of success included supportive supervision, project alignment with student interests, and opportunities for skill development, while barriers such as social isolation, limited support, and time constraints significantly affected engagement and outcomes. Although these findings are from a single institutional context, the identified themes are consistent with international literature and may therefore be relevant to other medical programs that integrate research as a core component. Ultimately, they highlight the importance of deliberate, structured support and inclusive program design to ensure that research education is both effective and equitable across diverse educational settings.

## Data Availability

The datasets generated and/or analysed during the current study are not publicly available, but deidentified data are available from the corresponding author on reasonable request.
